# Novel ASD device closure for treatment of post esophagectomy leak: a case series

**DOI:** 10.1093/jscr/rjaf1053

**Published:** 2026-10-22

**Authors:** Nilesh Fernandopulle, Githma Wimalasena, Deedat Safeer, Randima Nanayakkara, Janaka de Silva

**Affiliations:** Department of Surgery, Faculty of Medicine, University of Colombo, 25 Kynsey Road, Colombo 00800, Sri Lanka; University Surgical Unit, National Hospital of Sri Lanka, Colombo 00700, Sri Lanka; Department of Surgery, Faculty of Medicine, University of Colombo, 25 Kynsey Road, Colombo 00800, Sri Lanka; University Surgical Unit, National Hospital of Sri Lanka, Colombo 00700, Sri Lanka; Department of Surgery, Faculty of Medicine, University of Colombo, 25 Kynsey Road, Colombo 00800, Sri Lanka; The National Cancer Institute, Maharagama, Maharagama 10280, Sri Lanka; Faculty of Medicine, University of Kelaniya, Kandy Road, Kelaniya 11600, Sri Lanka

**Keywords:** ASD device, therapeutic endoscopy, anastomotic leaks

## Abstract

Anastomotic leaks occur in 5%–29% of patients after esophagectomy for malignancy and remain a major therapeutic challenge. Conventional options include surgical repair, diversion, endoscopic, or conservative management. A novel approach uses an atrial septal defect (ASD) closure device—originally designed for cardiac use—to seal post-esophagectomy leaks. We report four male patients who developed anastomotic leaks within 14 days after laparoscopic esophagectomy for gastroesophageal junction tumors. Standard management failed, and ASD device placement achieved successful closure in three cases, with long-term follow-up available for two. This series highlights the feasibility, safety, and efficacy of ASD devices as a minimally invasive option for refractory esophageal leaks. Although experience remains limited, these devices show promise as an alternative or adjunct to conventional methods. Further studies comparing outcomes and durability are needed to define their role in gastrointestinal leak management.

## Introduction

Oesophagectomy is a surgery which carries several post operative complications, anastomotic leakage being one of the most serious. It has been shown to occur in 5%–29% patients undergoing the procedure [1]. Several treatment strategies are employed to correct the defect, which conventionally include conservative management, surgical repair, diversion and exclusion, and endoscopic procedures. Endoscopic procedures include endoscopic suturing, endoscopic clip insertion, stenting, and use of fibrin glue [2, 3]. A novel method of treating anastomotic leakage following oesophagectomy is the use of an atrial septal defect (ASD) closure device to seal off the leakage site. This technique was first reported in two case reports published in 2010 by Green *et al*. and Repici *et al*. [4, 5]. All case reports published regarding this procedure have used the Amplatzer Septal Occluder Device, which is a double umbrella shaped device, made of a nitinol wire mesh, and is self-expanding [3–9]. This structure of the closure device promotes tissue in-growth and closure of leakage sites and fistulas and is available in a range of waist and disk sizes, being ideal for endoscopic treatment of anastomotic leakage despite it being an off-label use of the device [10]. Of the available case reports, migration of the occlusion device was reported in one case report [7], and 4 case reports had successful outcomes during follow up to a maximum of eight months [3, 5, 6, 8].

We report a case series of four patients who underwent closure of their post esophagectomy anastomotic leaks with ASD devices and followed for 1–36 months (Table 1).

**Table 1:** Patient characteristics, defect size, ASD device details, follow-up, and outcomes following post-esophagectomy leak closure.

	Diagnosis	Age (yrs)	Size of defect (mm)	ASD size (mm)	Follow up (months)	Complications
Case 01	GOJ Adeno Ca	75	7	12	24	None
Case 02	GOJ Adeno Ca	20	10	12	36	Needed repositioning
Case 03	GOJ Adeno Ca	47	14	16	6	None
Case 04	GOJ Adeno Ca	56	20	22	1	None

GOJ: Gastroesophageal junction.

Ca: Carcinoma.

## Case discussion

Anastomotic leak was clinically suspected and confirmed by contrast enhanced computed tomography (CECT) during the 2^nd^ post operative week in all four patients. Three patients were referred immediately after the diagnosis, and one was conservatively managed and referred 4 weeks after surgery due to non-resolution of the leak. The three patients referred during the 2^nd^ post operative week had evidence of sepsis with hemodynamic compromise, and were on parenteral antibiotics, and intercostal tubes.

Conventional methods such as over the scope clip (OTSC) and esophageal fully covered metal stent were used initially without success in two patients. Afterwards the patients were referred to the first author for further management of the leak.

The occluding device (Amplatzer Occluder; AGA Medical Corp, Plymouth, Minn) consists of a nitinol wire mesh – self-expandable and double umbrella-shaped (Fig. 1). There is a connecting constrainable waste between the two umbrellas. Polyester fabric is securely sewn to each disk internally to help close the hole after placement. The size of a device is identified by the diameter of the waste, which would approximately correspond to the size of the defect we need to close. The device can collapse to a linear shape by pulling from both ends. This helps to load the device into the scope before application. There are radio opaque markers for use during fluoroscopy.

**Figure 1:**
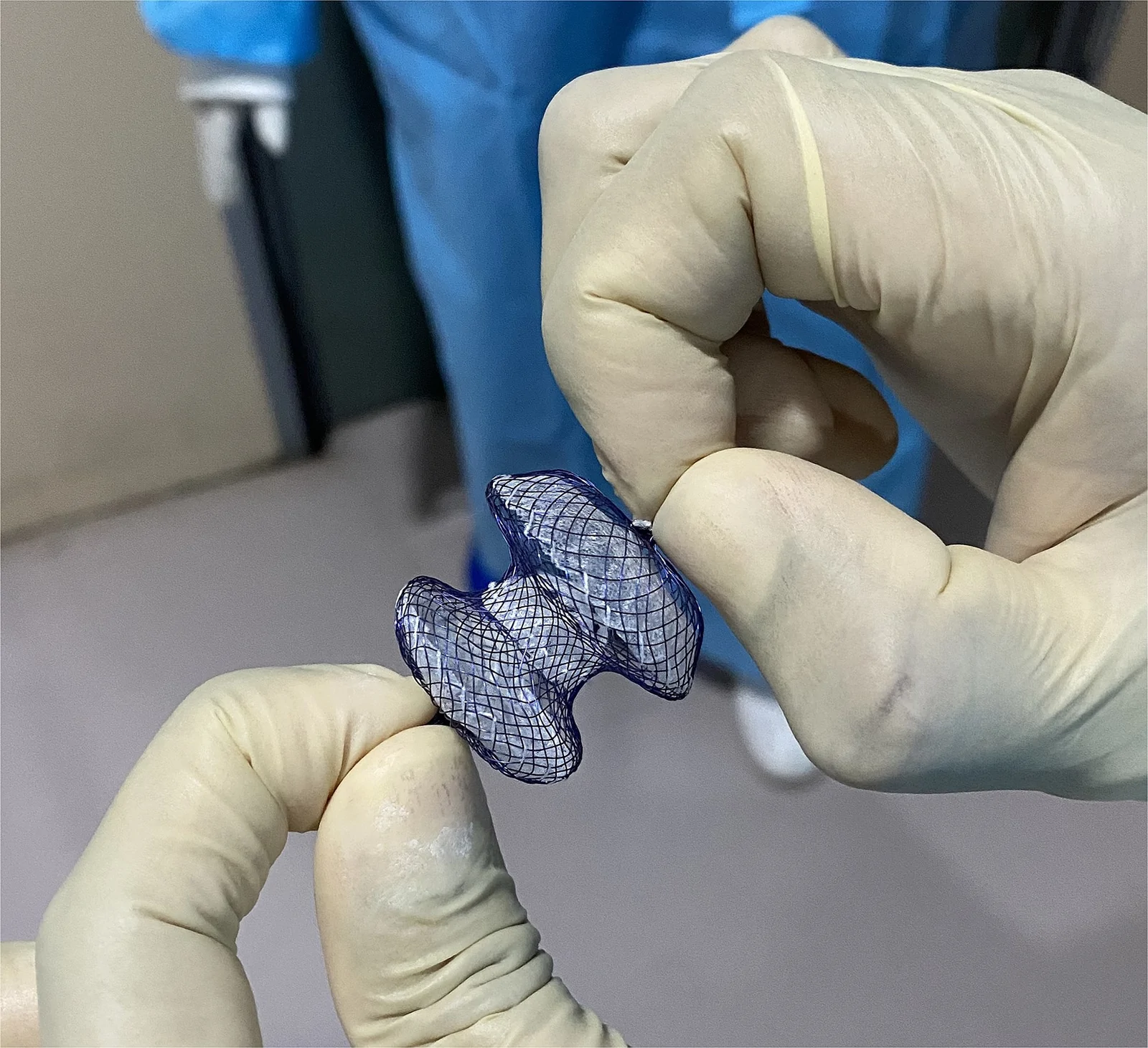
Atrial septal defect (ASD) closure device.

The procedure was performed with the patient under conscious sedation, using a standard adult gastroscope. An initial endoscopy was done to assess the size and site of the leak (Fig. 2). All leaks were within the anastomotic site, with the circular staplers seen adjacent to it. The approximate size of the defect was measured using a biopsy forceps. CO_2_ insufflation was used during the assessment. One patient’s oxygen saturation dropped and needed urgent intercostal tube insertion to improve ventilation, after which the procedure was continued.

**Figure 2:**
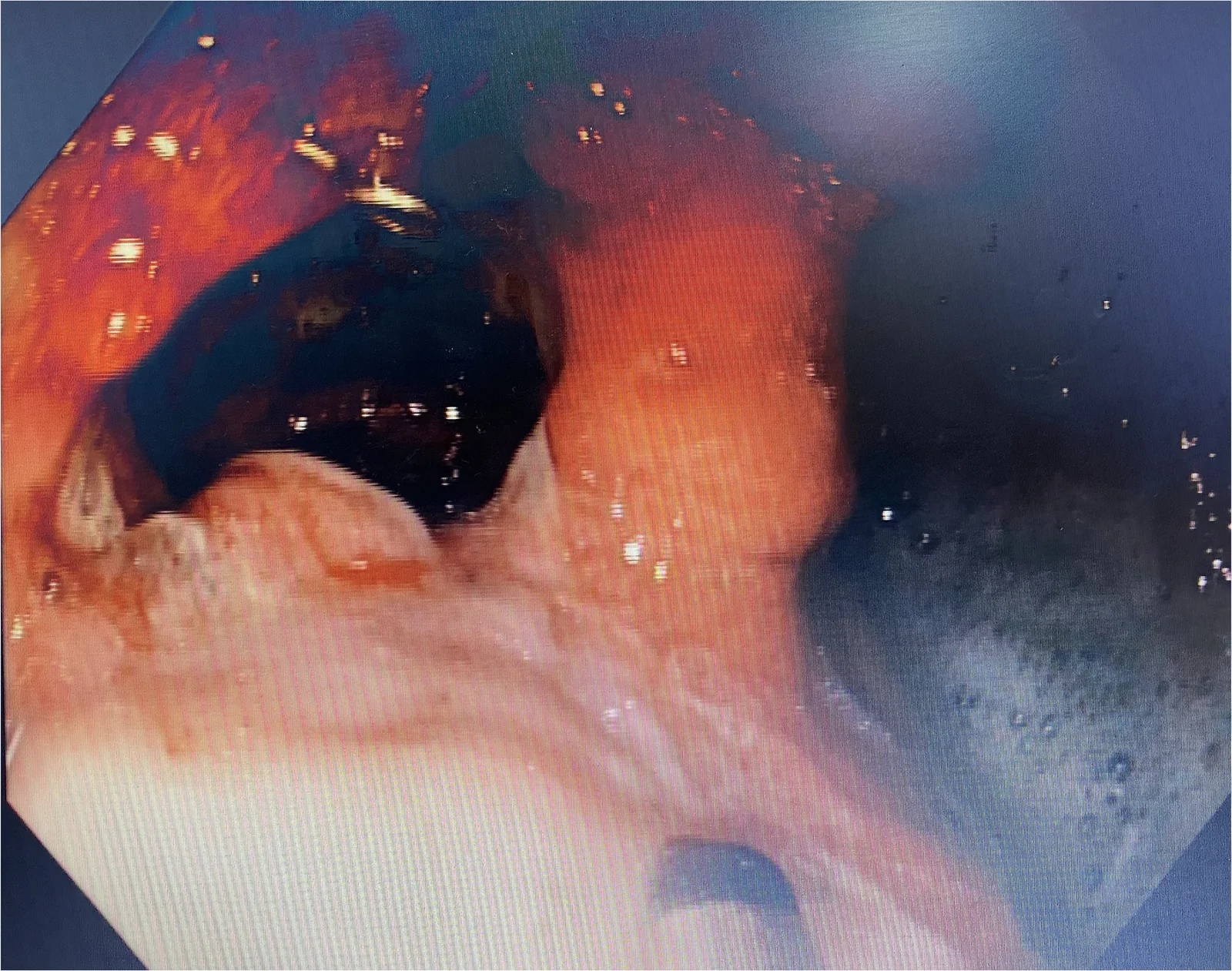
Endoscopic view of the anastomotic leak/defect site.

Initially a pediatric biopsy forceps is introduced through the gastroscope accessory channel, and the top end of the ASD device is grasped with the forceps and pulled back into the accessory channel, so the device collapses within the channel. An adult biopsy forceps cannot be used as the forceps arms which a partially closed when held with the ASD device cannot be pulled back into the accessory channel. When using the large 22 mm ASD device we had to use a pediatric colonoscope as the larger device would not collapse into the gastroscope channel. An ASD device size 2-4 mm bigger than the defect size is used to close the defect (Fig. 3). After loading the device into the scope. The scope is advanced into the external cavity to the defect and the distal umbrella is pushed across the defect into the potential space seen outside of the esophagus, and the distal umbrella is opened out within that external space. It must be understood that due to the limited space, the distal umbrella may not open completely to achieve an umbrella shape. Then the scope is pulled back so that the mid-section of the device is aligned to the esophageal wall at the anastomotic leak site and the proximal umbrella is delivered within the esophageal lumen while withdrawing the scope. The proximal umbrella takes the shape of a flat disc and firmly sits on the leak covering the opening in the esophageal side. This may have to be repeated a few times before a perfect position is found. The ASD device can be pulled out from the leak site and replaced until this perfect position is found and the leak appears to be well sealed by the disk (Fig. 4). An ASD device which is 2–4 mm larger than the size of the defect would tightly position at the leak site and oppose the two sides of the defect opening. An endo clip can be applied to catch the mesh of the ADD with the adjacent mucosa. This minimizes dislodgement of the device toward the esophageal lumen. A draining naso gastric tube placed to remove in secretion from the gastric cavity and feeding is continued via the feeding jejunostomy created surgically. After one week a contrast study is done to confirm absence of a leak (Fig. 5a and b). Oral feeding started 2 weeks after placement of the device.

**Figure 3:**
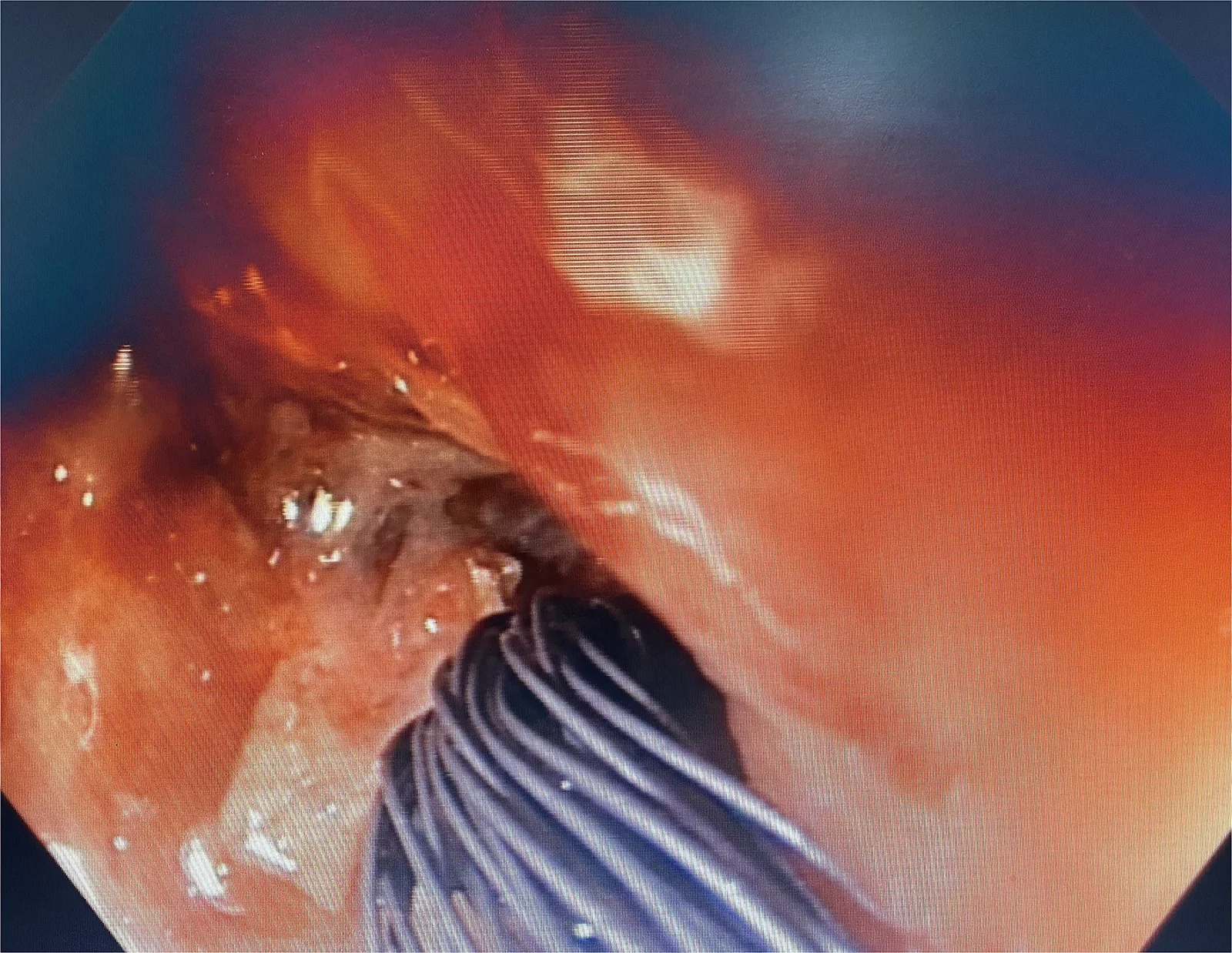
Endoscopic visualization of the ASD closure device positioned at the leak site.

**Figure 4:**
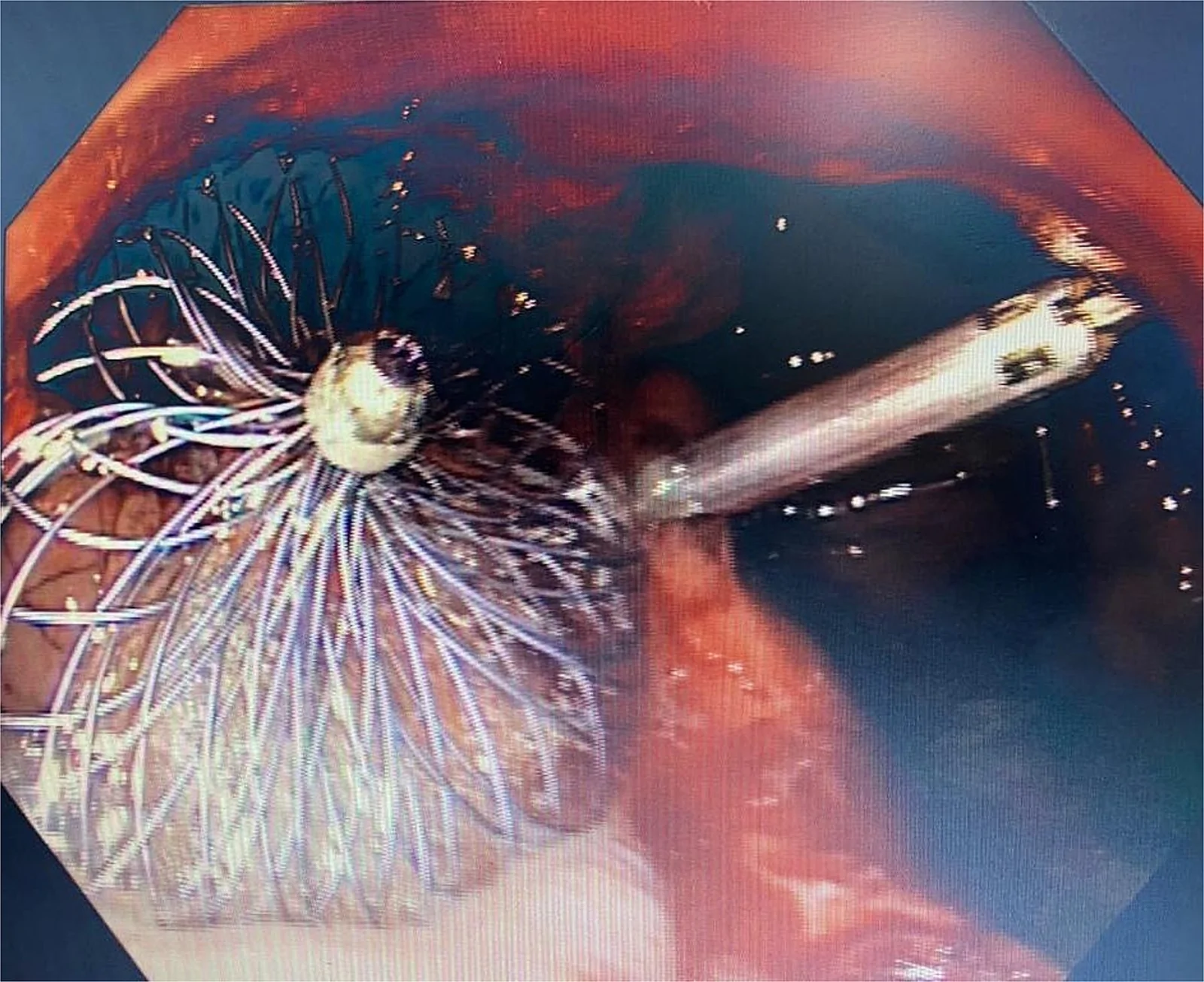
Endoscopic view confirming secure placement of the ASD closure device.

**Figure 5:**
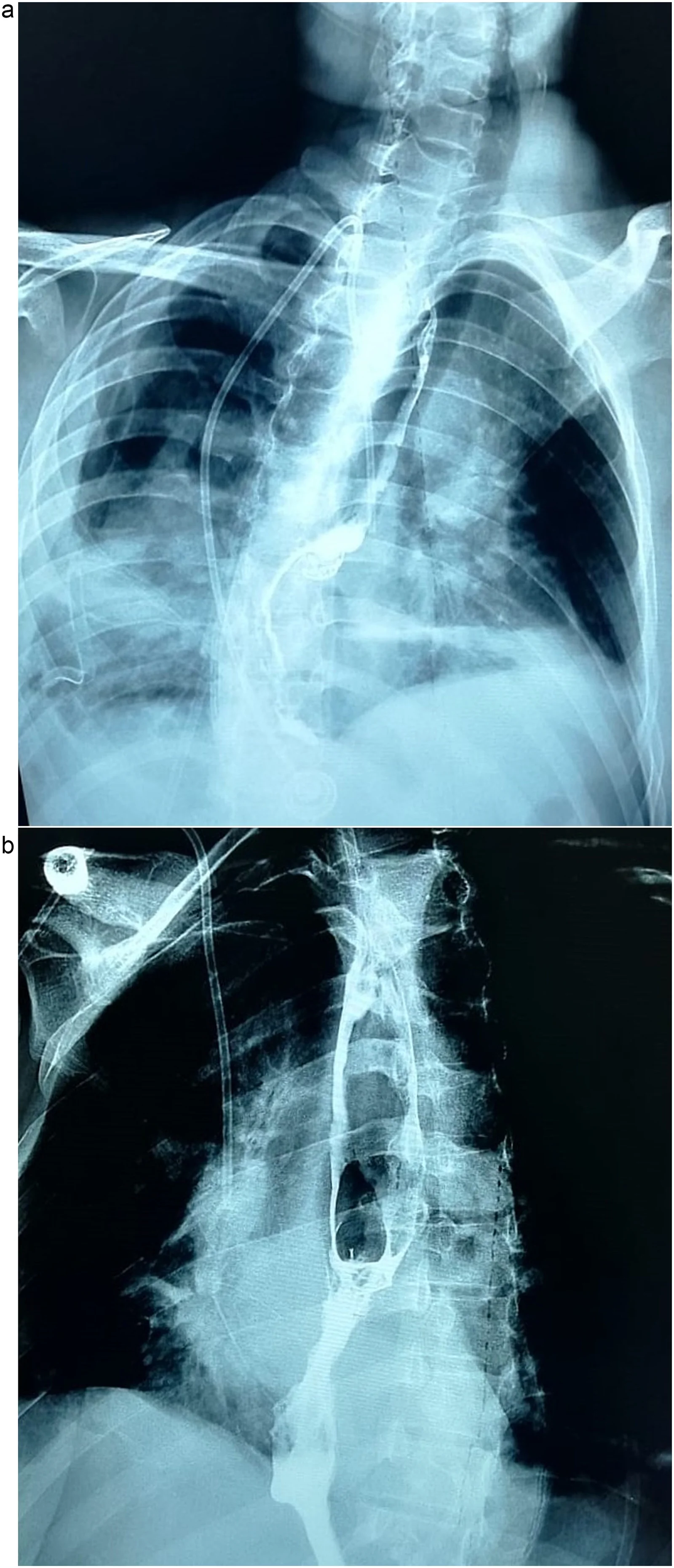
(a) Post-procedure contrast swallow study (anteroposterior view). (b) Post-procedure contrast swallow study (lateral view).

In three patients, CECT done 6 months post operatively showed the ASD device dislodged into the stomach in two patients and at the site in one patient and Oesophagogastroduodenoscopy (OGD) done at the same time confirmed the CT findings and the site of the anastomotic leak had completely closed off. The two devices free in the stomach were removed with a snare The other device still at the leak site was left in situ and there was no mucosal defect seen. The fourth patients ASD device had dislodged into the esophageal lumen after 2 weeks. As this patient was transferred back to the original unit after the procedure, we could not re-intervene, and the primary surgeon had removed the device and decided to manage the patient conservatively as patient had developed seizures and post-surgical radiological imaging suspected brain metastasis.

## Discussion

Case reports on the use of ASD closure devices in gastrointestinal tract related interventions are limited worldwide. The use of ASD closure devices for correction of duodenocutaneous fistula preceded that of correction of anastomotic leakage after oesophagectomy and was first reported in 2009 by Boulougouri *et al*. [11]. Subtil *et al*., Coppola *et al*., and Repici *et al*. have reported the use of this device for closure of tracheoesophageal fistulae [5–7], and use of similar device for closure of other gastrointestinal fistulae have later been reported [3, 4, 8, 9].

Healing of the defect and resolution of the leak has been evaluated via CECT in several studies, as well as through endoscopy. When used in cardiac surgery to close cardiac wall defects, ASD closure devices are designed with a surface coating which promotes neoendothelialization. Case reports from Milan, Italy, and Pamplona, Spain have reported mucosal epithelialization over the ASD closure device with healing of the defect noted on endoscopy [5, 6], when used to close defects in the gastrointestinal tract. In a case report from Italy, where an ASD closure device was used to close a tracheoesophageal fistula, the migration of ASD device resulted in failure of the procedure and put patient at risk of airway obstruction [7].

Interestingly unique to two of our patients and quite contrast to the literature, the device had completely dislodged toward the esophageal side at 6 months, and the leak was completely closed. Therefore, we suspect the device has worked as an occlusion to seal the defect and the epithelialization had happened from the outside of the device (external to the distal umbrella), unlike in previous literature where the epithelialization had occurred over the proximal end fully submerging the device. In our case series, as the patients were clinically well, we waited for 6 months to repeat imaging and OGD, but it’s very likely the healing would have taken place much quicker and follow-up studies would be useful to propose timelines and protocols for post ASD device placement follow up.

Published series comparing endoscopic options for post-esophagectomy leaks suggest that endoscopic vacuum therapy (EVT) achieves leak closure in a shorter time and with fewer re-interventions than stenting [12]. Median treatment/closure times with EVT are typically ~15–23 days with ~4–6 sponge exchanges [13], whereas stent-based therapy commonly requires longer indwell (~4–6 weeks) and more frequent unplanned endoscopic re-interventions, largely due to migration. In comparative cohorts and meta-analyses, EVT shows higher clinical success and lower complication rates [14]; reported stent migration ranges from ~17% to >30% depending on device and pathology [15, 16], with re-intervention rates higher than with EVT. Although there is no comparative data, ASD device is cheaper, has no discomfort to the patient as seen after placement of an EVT and has less reintervention rates compared to stenting, and may even achieve higher success rates.

The cases included in our report were referrals from multiple surgical units rather than a single institutional cohort; therefore, the overall number of esophagectomies and anastomotic leaks across units would not be representative of the volume in one center. At present, we do not maintain a consolidated database tracking total esophagectomy volumes and associated leaks across all referring units for formal analysis.

The use of ASD closure devices in the management of gastrointestinal fistulas and anastomotic leakage provides a minimally invasive method of correcting the defect, with higher success rates than alternatives such as stent insertion [8]. The unique structure of the ASD closure device which promotes tissue in-growth, and the availability of multiple disk and waist sizes allows the device to close the defect with irregular margins, edematous, and scarred margins which pose difficulties when managing such defects using clipping, suturing, and stenting [10]. This method also can be used in any patient irrespective of age, body habitus, and other co morbidities. It’s also a relatively simple procedure in experienced hands and can be performed with conscious sedation. Given these added advantages, we propose that ASD closure devices should be considered as an effective and viable option to manage post operative gastrointestinal defects.
